# Tomato Genomic Resources Database: An Integrated Repository of Useful Tomato Genomic Information for Basic and Applied Research

**DOI:** 10.1371/journal.pone.0086387

**Published:** 2014-01-21

**Authors:** B. Venkata Suresh, Riti Roy, Kamlesh Sahu, Gopal Misra, Debasis Chattopadhyay

**Affiliations:** National Institute of Plant Genome Research, Aruna Asaf Ali Marg, New Delhi, India; Nanjing Forestry University, China

## Abstract

Tomato Genomic Resources Database (TGRD) allows interactive browsing of tomato genes, micro RNAs, simple sequence repeats (SSRs), important quantitative trait loci and Tomato-EXPEN 2000 genetic map altogether or separately along twelve chromosomes of tomato in a single window. The database is created using sequence of the cultivar Heinz 1706. High quality single nucleotide polymorphic (SNP) sites between the genes of Heinz 1706 and the wild tomato *S. pimpinellifolium* LA1589 are also included. Genes are classified into different families. 5′-upstream sequences (5′-US) of all the genes and their tissue-specific expression profiles are provided. Sequences of the microRNA loci and their putative target genes are catalogued. Genes and 5′-US show presence of SSRs and SNPs. SSRs located in the genomic, genic and 5′-US can be analysed separately for the presence of any particular motif. Primer sequences for all the SSRs and flanking sequences for all the genic SNPs have been provided. TGRD is a user-friendly web-accessible relational database and uses CMAP viewer for graphical scanning of all the features. Integration and graphical presentation of important genomic information will facilitate better and easier use of tomato genome. TGRD can be accessed as an open source repository at http://59.163.192.91/tomato2/.

## Introduction

Tomato (*Solanum lycopersicum*) is model for a number of biological studies important to agriculture such as, fruit development and ripening, disease resistance and biochemical pathways of important nutrients [1]–[3]. Tomato, a *Solanaceae* member, has been used for pioneering research on cell wall and storage polysaccharide synthesis and degradation [4]–[6]. Tomato was the first plant from which a ‘gene for gene’ class of R-gene for disease resistance was cloned [7]. Tomato fruits are a rich source of carotenoid pigment, which is the precursor of vitamin A. Tomato has been used extensively for genetic studies because of several reasons such as, its diploid genome, short generation time, availability of homozygous inbred lines, efficient transformation technology and its genes are largely sequestered in contiguous euchromatic regions [8]–[10]. Apart from basic research, tomato is the second most-consumed vegetable in the world. Researchers from fourteen different countries together have published a reference genome sequence of an inbred tomato cultivar Heinz1706 and a draft sequence of wild tomato, *Solanum pimpinellifolium* [11]. These sequence information have provided the basic and applied researchers an opportunity to scout for gene function, genetic diversity and evolution not only in tomato, but also in other *Solanaceae* members, for studying basic biology and bio-diversity based breeding. The Sol genomics network (SGN) serves as the most referred host for storing and integrating most of the information on *Solanaceae* crops and is continuously developing [12]. There has been existence of a number of other tomato databases on specific aspects even before the release of tomato genome such as, SolEST database, MoTo DB, Tomato functional genomics database, miSolRNA, TOMATOMA, KaTomicsDB (http://www.kazusa.or.jp/tomato/) and several others [13]–[18]. These databases serve extensively as resources for genomic and biochemical information and biological material for the *Solanaceae* research community. However, there is a need to amalgamate different genomic and trait information together graphically along all the chromosomes of tomato in a single window to enable the basic and applied researchers to integrate and utilize all the information in a better and easier way.

Microsatellite markers (simple sequence repeats, SSRs) are important for various applications such as construction of high-density linkage maps, comparative genome mapping, identification of variety, marker-assisted selection, studying genetic diversity and so on. They are reliable because of high experimental reproducibility, multiallelic nature and co-dominant inheritance [19]. Simple sequence repeats (SSR) are the most popular microsatellite markers. Genes are the most important functional part of a genome and define the trait of an organism. Integration of SSRs with genes in a single window will allow the researchers to look for presence of SSRs in the coding, non-coding and the upstream activating sequences of a gene and subsequently for intra- and inter-species polymorphism. Genetic variation of a trait within a species can be attributed to a single gene or joint action of many genes that can be mapped on the genome (QTL, Quantitative trait loci) by genetic markers [20]. Integration of well-known QTLs with the genes and SSRs along chromosomal length will provide the users an easier access to the genes and SSRs co-localizing with a QTL. Changes in gene expression may lead to phenotypic differences between two individuals. Apart from transcriptional regulation, gene expression can be regulated at various stages including through microRNAs (miRNAs). Several studies demonstrated that development and metabolism of plants are regulated by miRNAs [21]–[23]. miRNAs might also have potential role in transgressive phenotype [24]. Therefore, inclusion of tissue-specific expression of genes, miRNAs and their predicted target genes together with SSRs, genes and QTLs would be useful. Small and red-fruited *Solanum pimpinellifolium* is the closest wild relative of domesticated tomato. Introgression lines for *S. pimpinellifolium* in the background of cultivated tomato are used for mapping different traits. There is a 0.6% nucleotide divergence between the inbred cultivar Heinz1706 and the *S. pimpinellifolium* accession LA1589. This huge resource of SNPs, especially in the genic region, would immensely benefit the breeders to utilize the natural trait reservoir for crop improvement. In view of above, we have integrated SSRs, genes, miRNAs, known QTLs and SNPs between the sequenced cultivated and wild tomato accessions in a single window along the length of all the twelve chromosomes of tomato.

## Materials and Methods

### Sequence Retrieval

S. *lycopersicum* Heinz 1706 reference genome sequence, raw sequence reads of *S. pimpinellifolium* LA1589, latest annotation (ITAG 2.3), and gff files were downloaded from Sol genomics network database (SGN) (www.solgenomics.net). The RNAseq data was downloaded from short read archive of NCBI [accession number SRX118613 (leaf) SRX118614 (root) SRX118615 (flower) SRX118621 (mature green fruit)]. QTL and EXPEN-2000 map coordinates were retrieved from SGN.

### Microsatellites/SSRs

MIcroSAtellite identification tool, MISA (http://pgrc.ipk-gatersleben.de/misa/) was used to scan the tomato reference genome with default parameters to identify repeats. MISA program allows the user to specify the minimal length of the consecutive nucleotide repeat and reports the SSR type, SSR motif, motif repeat, length of repeat and coordinates of the SSRs in the genome. Mononucleotide repeats were not included. The minimum number of repeats allowed for the dimers was six and for tri-hexamers was five. Primer3 program was used to design the primers from the flanking regions within 100 bp of each of the identified SSR [25]. Coordinates for all the genes were retrieved from the gff file of ITAG2.3 annotation. Coordinates and sequence of 2 kilobase (kb)-long upstream sequences (5′-US) of the genes were extracted with PERL script. Accordingly, SSRs were assigned to 5′-US and different parts (exon, intron, and 3′ untranslated region) of the genes in addition to other parts of the genome. All the SSRs, for which primers could be designed, were anchored on to all the twelve chromosomes and can be visualized interactively using CMAP viewer [26].

### SNPs and miRNAs

Raw Illumina reads of *S pimpinellifolium* were filtered for high quality through NGS QC Toolkit v2.3 with default parameter [27]. Paired end reads were mapped on tomato reference genome through Bowtie version 2.1.0 with default parameters [28]. SAMTools was used to convert SAM file to BAM file, remove duplicate reads and for SNP calling [29]. Stringent SNP filtering criteria of minimum read depth 5, minimum root-mean-square mapping quality 30 and all the mapped reads showing same non-allelic base were applied. SNPs in the protein-coding genes were extracted using gene coordinates and accordingly assigned to the different parts of the genes as mentioned above. MicroRNAs with their targets were retrieved based on the available literature and from miRBase database, and the co-ordinates were retrieved from.gff file of reference genome [30], [31], [11].

### Genetic Markers and QTLs

Genetic markers and QTLs were retrieved from EXPEN-2000 and QTL genetic maps, respectively, from SGN database and literature (*Ty1/3, Pto, Bs4, Cf-9, Ve, Mi*) [32]–[37]. All the EXPEN-2000 genetic markers were anchored to the chromosomes according to their physical locations. All the QTLs were assigned according to the physical locations of the nearest flanking sequence-characterized genetic markers mentioned in SGN. Where the sequences of the flanking markers were not available, physical coordinates of the corresponding EXPEN2000 genetic positions, if available, were retrieved from Kazusa Tomato Marker Database (http://www.marker.kazusa.or.jp/Tomato/) for assigning to chromosomal locations.

### Gene Expression

RNA sequence data for four tissues, namely leaf (3 week-old), root (3 week-old), flower (unopened bud) and mature green fruit, of tomato cv. Heinz1706 was retrieved as mentioned above and used for the analysis. Illumina RNAseq reads were first aligned to ribosomal RNA sequences using Bowtie 2 to eliminate possible rRNA sequence contamination. To quantify the expressions of genes all the Illumina reads from 4 tissue samples were mapped onto the genes using RSEM [38]. The number of reads mapped was normalized by RPKM (reads per kilobase per million) method. The heat map showing gene specific expression was generated on the RPKM for each gene in all the tissue samples using TIGR MultiExperiment Viewer [39], [40]. Differential gene expression analysis was performed using DESEQ [41]. The genes showing greater than two fold expression with p≤0.05 were regarded as differentially expressed. Genes expressing with more than 5 RPKM in a tissue as opposed to less than 1 RPKM in other tissues were considered as preferentially expressed. Genes preferentially expressed in each tissue sample, as compared with others, in a tissue-by-tissue comparison is presented as heat map. To find out collinear gene blocks on tomato genome, all-versus-all BlastP match was performed using the tomato proteome (version: ITAG2.3) with cut off e-value of 1e^−5^. The BLASTP result was fed into MCScanX toolkit (http://chibba.pgml.uga.edu/mcscan2/) to identify collinear blocks with the following parameters; e-value of 1e^−5^ and match size of 10. A total of 773 collinear blocks were detected with 20 genes as a minimum block size.

### Structure of TGRD

**Figure 1:**
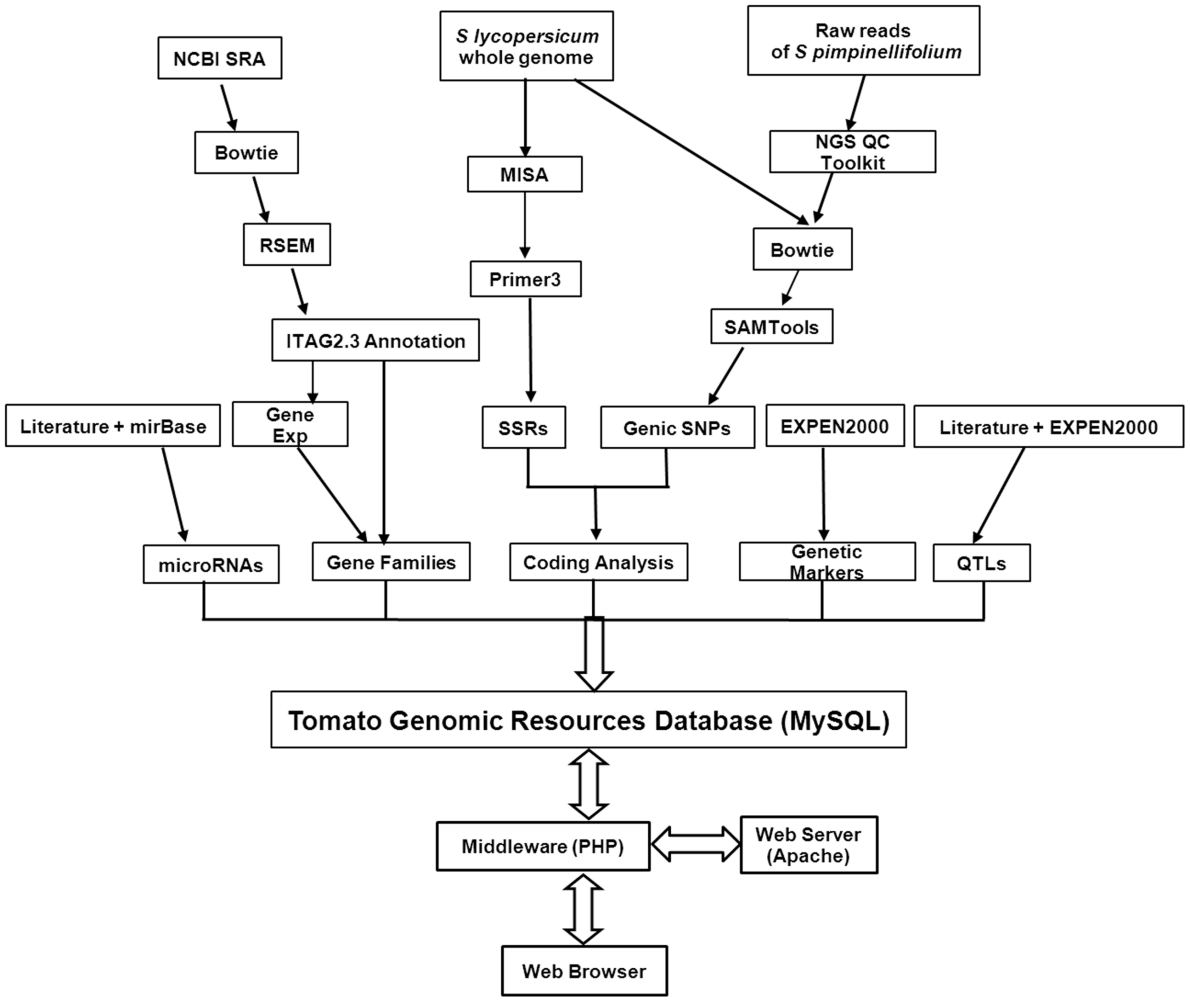
Schematic illustration showing the flow of the organization of the data in tomato genome database.

The Tomato Genomic Resources Database (http://59.163.192.91/tomato2/) is an online and interactive relational database developed using open source software, Apache (http://www.apache.org), MySQL 5.0 (http://www.mysql.com), JavaScript and PHP 5.4 (http://www.php.net) and is hosted on a 64-bit Linux Server. The database was designed based on ‘Three-Level Schema Architecture’ (figure S1). A flow chart explaining the database architecture, organization and workflow of TGRD has been presented in figure 1. The CMAP viewer and its schema were integrated with TGRD for graphical scanning and analysis of various genomic features. The user-friendly interface for TGRD has been developed using PHP 5.4, JavaScript and HTML to query and retrieve the data based on user needs. The interface is completely interactive and interlinked with each component of the genome. For example, the gene page contains information about the associated SSRs and SNPs; and the genic SSR page contains information about the gene, which contains the SSR and so on. The TGRD database contains a robust graphical tutorial to facilitate better use of the database.

## Results and Discussions

### Sequence Based Physical Mapping

**Figure 2:**
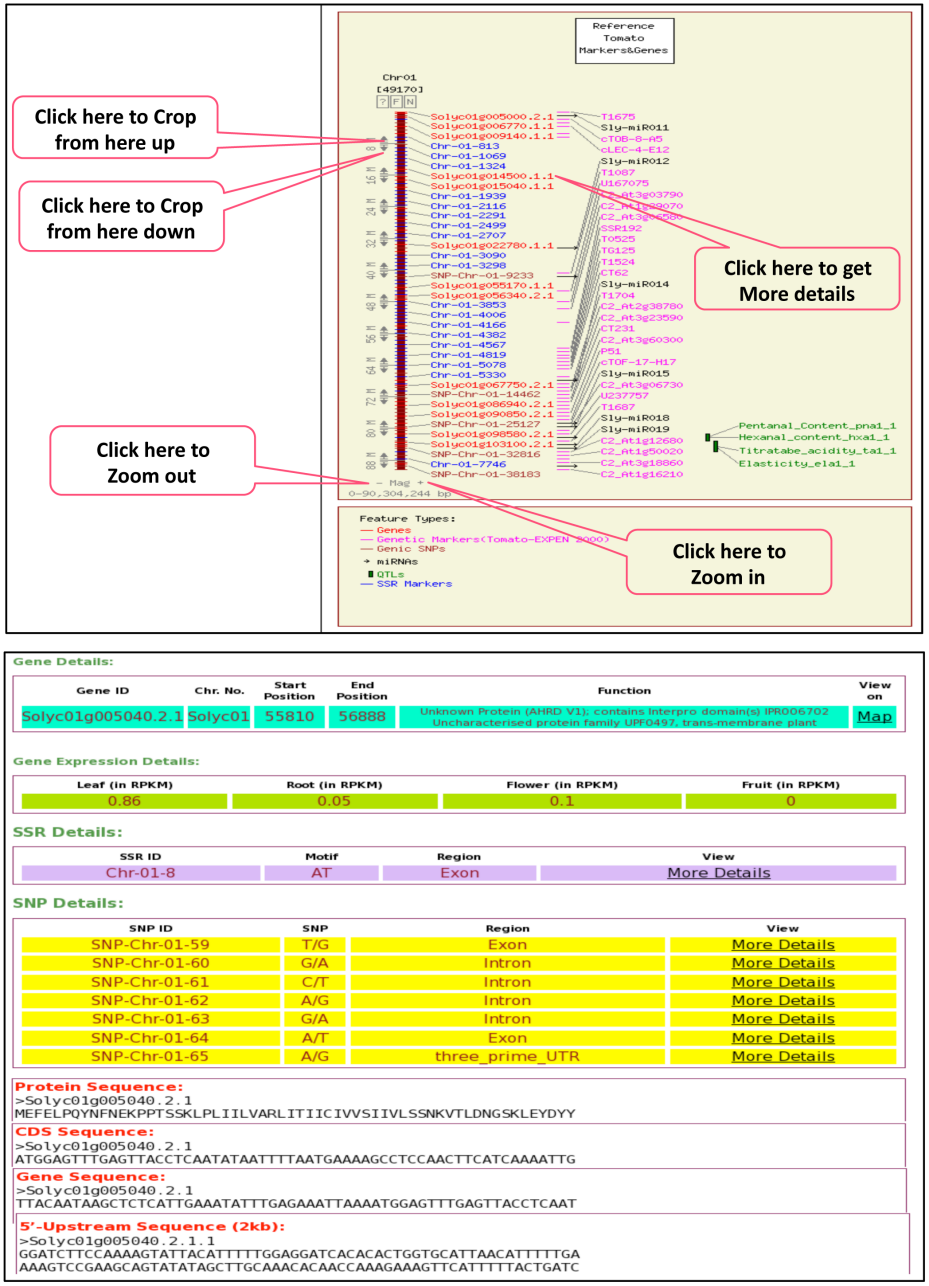
Screenshots of a map view and a gene description page showing various features of tomato genome database.

All the genomic features, eg. genes, SSRs, genic SNPs, QTL, miRNAs and genetic markers (EXPEN-2000) are mapped on twelve chromosomes and visualized by using CMAP viewer. The CMAP viewer can be accessed from “MAPs” tab. The MAP page is developed with rich interactivity using JavaScript and HTML. It shows all the twelve chromosomes in circular form with interactivity. The user can visualize all the twelve chromosomes together (‘All Chromosomes’ tab at the centre) or individually with all or individual genomic features to have better comprehension about tomato genome. On CMAP viewer, each genomic feature contains links to get further information. The CMAP viewer contains ‘Feature’, ‘Display’ and ‘Advanced’ options at the bottom to control and reconstruct the map to view the features on the physical map as per user’s option. For better utilization of space, the overlapping features were collapsed. Choosing ‘No’ option for ‘Collapse Overlapping Features’ in ‘Feature’ option would separate the features. Different features are color-coded. The maps can be cropped and magnified for any particular region of the chromosome to see the detail features. EXPEN-2000 genetic markers are included because they are well known to the breeders and, therefore, the other feature can be viewed with respect to the positions of these markers. EXPEN-2000 markers are linked to SGN site for detail information. Examples of two CMAP viewer pages with all the genomic features are presented in figure 2.

### Simple Sequence Repeats

**Figure 3:**
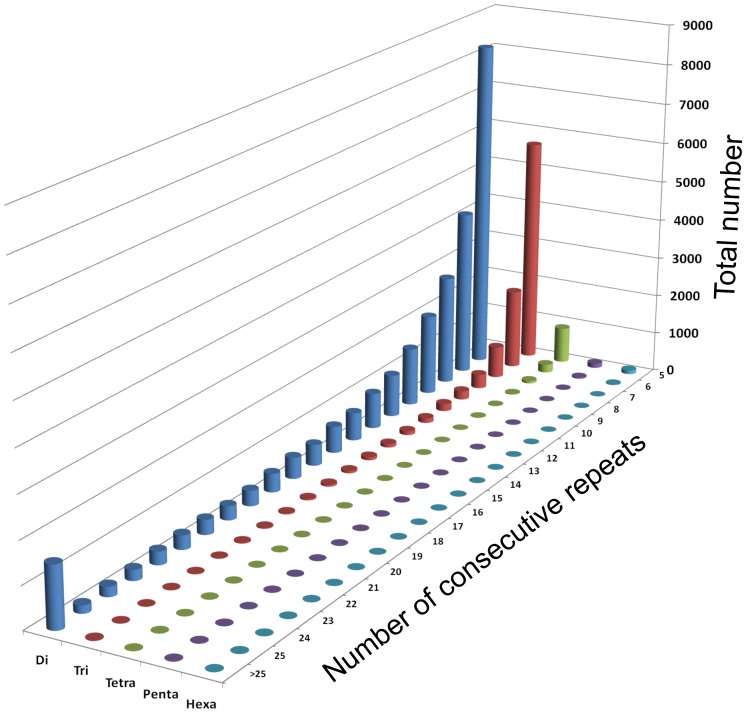
Relative frequency and number of selected microsatellite repeat-motif types in tomato genome.

We mined the reference genome of tomato for microsatellite repeats. Total 68,641 microsatellite repeat motifs spanning 781 Mb assembled genome were identified. Dinucleotide repeats (60.18%) are much more abundant than tri (19.56%) and other repeats. Scaffolds assigned to twelve chromosomes spanning 760 Mb contain 66,823 sequence repeats of which, 55,396 (82.90%) and 11,427 (17.10%) were simple and compound repeats, respectively. Of the 55,396 simple repeats, primer pairs for 38,659 SSRs (69.78%) could be successfully designed. Frequency and distribution of various SSRs were analyzed. Chromosome-wise repeat statistics is presented in figure 3 and table S1. All the SSRs, for which primer pairs were designed, were anchored to individual chromosomes according to their coordinates. Physical locations of the SSRs in the exon, intron or 2 kb 5′-upstream regions of the genes were assigned. The user can query the database from ‘SSRs’ tab using motif sequence, chromosome number, two coordinates of a chromosome or combination of repeat length and minimum number of repeat. The SSRs present in the gene coding (Genic SSRs) or in the 5′-upstream sequences of the genes (5′-US SSRs) also can be searched. Total of 5841 and 4773 SSRs were present in 33,840 chromosome-assigned genes and their 5′-upstream sequences, respectively, with average frequencies of 0.172 SSRs/gene and 0.14 SSRs/5′US. The query results information on marker ID, motif sequence, motif length, motif repeat, repeat length, region, chromosomal location, start and end positions, functional information in case of genic SSRs, primer information and link to CMAP to view location on chromosomes for each entry present in the database. These SSRs, physically localized along the chromosomes, would serve as an immense resource for comparative genome mapping, genetic diversity study and identification of polymorphic marker for quantitative trait loci.

### Genes, Gene Families and microRNAs

According to ITAG2.3 annotation, tomato reference genome contains 34,727 protein-coding genes, of which 33,840 are assigned to twelve chromosomes so far. Chromosome 1 has the highest estimated (108.0 Mb) and assembled (90.30 Mb) lengths and accordingly, it codes for the highest number (4293) of genes. Whereas, the assembled chromosome 11 possesses the least (2385) number of genes, although its estimated (64.7 Mb) and assembled (53.4 Mb) lengths are more than the estimated (53.8 Mb) and assembled (46.04 Mb) lengths of the smallest tomato chromosome 6, which codes for 2813 genes [42], [11]. Each gene record provides information about chromosomal location, gene expression (RPKM values), sequence information (gene, CDS, protein, 5′-US) and associated SSRs and SNPs at different locations of a gene. Under the ‘GENE SEARCH’ tab, the user can search for any gene by ID, chromosome number, gene function and two coordinates of any chromosome. This tab is facilitated with BLAST search to look for any homologous tomato gene.

**Figure 4:**
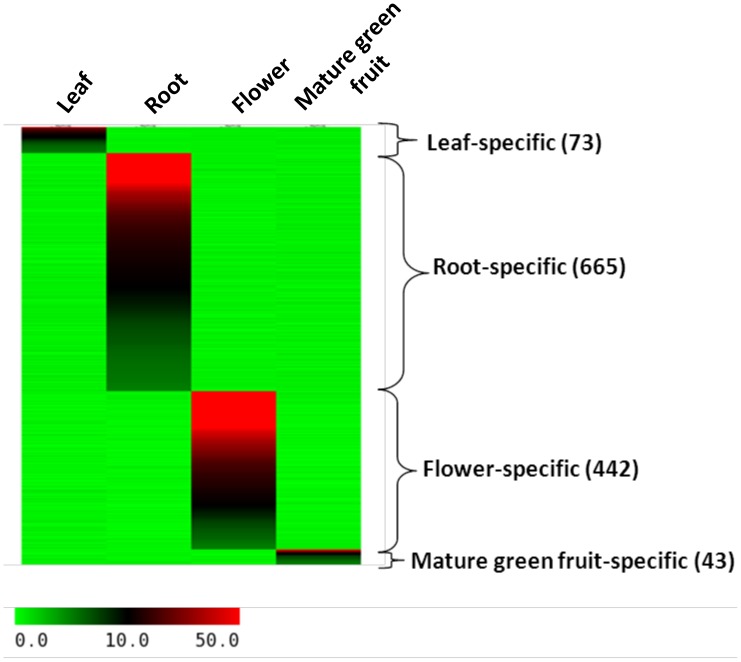
Heat map showing genes preferentially/specifically expressed in four tissue samples. The number of genes and tissue specificity is noted at the right side. The color scale (0–50) represents RPKM values.

**Figure 5:**
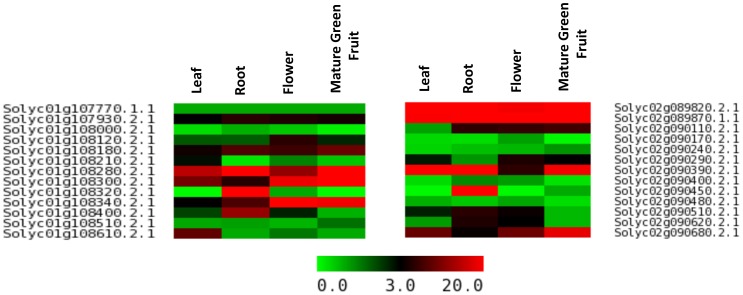
Differential expression profiles of genes present in two collinear blocks located on chromosome 1 and 2. The color scale (0–20) represents RPKM values. IDs of the genes in the collinear blocks in chromosome 1 and 2 are mentioned in the left and right sides, respectively.

Genes coding for transcription factors, heat shock proteins, protein kinases and transporter proteins are generally highly studied. Tomato has been extensively used as a model for disease resistance and fruit development. Apart from various transcription factors, enzymes and receptors for ethylene synthesis and perception, and red-light photoreceptors influence fruit development, ripening and quality. In addition, several enzymes related to modification of cell wall architecture have agronomic importance for fruit quality. Cytochrome P-450 family of genes involved in toxic alkaloid production showed significant contraction in tomato [11]. Therefore, genes encoding these protein families are specifically mentioned under the tab ‘GENE FAMILIEs’. All these genes can be viewed on CMAP viewer as a whole family or individually on all the twelve chromosomes. Total 2505 transcription factors assigned to the chromosomes are further subdivided into 89 families according to the domains present in them. Chromosome 1 possesses the highest number (287) of genes encoding transcription factors, while chromosome 9 contains the least (150). Similarly, 510 R genes are subdivided into eight families including the receptor like protein kinases. Chromosome 4 contains the highest number (85) of R genes. Functional genomics involves tissue-specific and treatment-specific expression of genes at different developmental stages of plants to predict function of a gene. We analyzed RNAseq data of tomato to reveal tissue-specific expression profiles of tomato genes. Hierarchical expression of all the genes in a family in leaf, root, flower and matured green fruit is provided in the form of heat maps and in RPKM values for individual genes. Total 6404 genes did not show any expression in these four tissues at these developmental stages. 73 genes were found to express preferentially in leaf while 665, 442 and 43 genes were found to express preferentially in root, flower and mature green fruit, respectively, at the specific developmental stage as mentioned (Fig. 4 and table S2). Physical positions of all the genes in a family can be viewed under this tab and, therefore, paralogous genes can be identified. Further, functional divergence of the genes present in collinear blocks can be analyzed from expression data as shown for two collinear blocks on chromosome 1 and 2 (Fig. 5). Integrated with QTL map, physical map of the genes provide an opportunity to associate candidate genes with important agronomic traits. 887 genes yet to be assigned to the chromosomes were listed with their detail under chromosome zero separately.

Out of 96 conserved miRNAs annotated in genome sequence, 93 have been assigned to twelve chromosomes and can be searched under ‘microRNAs tab. They are categorized in 26 families. As mentioned in the materials and methods, target genes for these miRNAs have been predicted based on literature and miRBase database. Each miRNA family contains information about their target genes, chromosomal location, strand orientation, mature and pre-mature RNA and DNA sequences.

### Single Nucleotide Polymorphisms (SNPs)

Cultivated tomato, which experienced a genetic bottleneck while carried from South America to Europe, has narrow genetic base with estimated <5% genetic variation in their wild relatives [43]. The wide genetic variation present in the self-compatible and –incompatible wild tomato species is being investigated and exploited in tomato improvement by introgression breeding. *S. pimpinellifolium* is used to study tomato traits such as fruit size and shape and to introduce bacterial spot resistance. High quality genomic sequence reads of *S. pimpinellifolium* were mapped to the tomato reference genome. Stringent filtering criteria were applied to extract highly probable SNPs between these two accessions. Total 2,971,025 high quality SNPs were obtained of which 290,445 are in protein-coding genes. Chromosome 7 and 12 possesses the highest (5361/Mb) and lowest (2551/Mb) frequencies of SNPs, respectively (table S3). Frequency distribution of genomic and genic SNPs and genes in each million base interval were plotted for each chromosome (figures S2 and S3) for better understanding of SNP distribution across chromosomes and especially in euchromatic regions. Except chromosome 12, heterochromatic regions of all the chromosomes showed higher sequence divergence than the euchromatic part (figure S2). Comparison of distributions of genes and genic SNPs (figure S3) identified ten regions of 1 Mb interval with twenty or more SNPs per gene and all these regions are in the low-gene heterochromatin regions of the respective chromosomes (table S4). A list of genes containing more than ten SNPs is listed in table S5. Most of them encode unknown proteins. Three genes encoding calcium-transporting ATPase, two encoding WD-repeat proteins and one encoding receptor-like protein kinase are among the annotated high SNP-containing protein-coding genes. All the genic SNPs are anchored to their respective locations in each chromosome and can be accessed directly from map or from the genes. Flanking sequences of 100-base length for each SNP are provided for designing primers to convert them as markers.

The genic SNPs identified can be utilized in finding allelic variation of known genes of economic importance and may initiate new studies. As an example, fruit size locus *fw2.2* is one of the QTLs selected during tomato domestication and accounts for as much as 30% of the difference between the fruit sizes of small-fruited wild *S. pennellii* and large-fruited domesticated *S. lycopersicum* [44]. *fw2.2* corresponds to a gene (Solyc02g090730.2.1, Chr 02∶46830407-46831197) that encodes a protein ‘Cell number regulator 1′ having structural similarity to human oncogene *Ras*. Of 42 SNPs between two alleles, only three SNPs change three amino acids within first nine residues of the protein. Therefore, the allelic variation in *fw2.2* was thought to modulate fruit size by differentially regulating carpel cell number due to differential expression of two genes rather than structural differences in proteins [45], [46]. Sequence comparison of Solyc02g090730.2.1 of domesticated Heinz1706 and another small-fruited wild *S. pimpinellifolium* LA1589 detected only two SNPs in the open reading frame. Of them, only one (SNP-Chr-02-23437), common for both *S. pennellii* and *S. pimpinellifolium*, changes the third amino acid. However, the 5′-upstream sequence (2 kb from translation start site), which regulates transcriptional expression of a gene, showed twenty one structural variations between Heinz1706 and LA1589, of which 15 are SNPs and six are in-dels (table S6). Significance of these allelic variations in regulating fruit size awaits experimental validation.

### Quantitative Trait Loci

Genetic mapping of a QTL allows researchers to look for candidate genes segregating around a trait locus, which ultimately results in QTL characterization. However, use of different mapping populations for QTL mapping hinders unified physical localization of multiple QTLs on a single physical map. We tried to anchor some QTLs to tomato chromosomes according to the physical locations of their flanking markers. We used QTLs described in SGN QTL map. This map is based on a mapping population derived from a cherry tomato line Cervil and a round large-fruited line Levovil, which was different from the mapping population (*S. lycopersicum* M82 X *L. pennellii* LA716) used for EXPEN2000 map [20]. Hence, the boundaries of QTLs could not be delineated accurately due to unavailability of sequence-characterized flanking genetic markers. The nearest sequence-characterized genetic markers or the corresponding EXPEN2000 genetic locations were, therefore, used to physically localize the QTLs, which resulted in assignment of a little wider physical length than the actual. Following this approach we could localize fifty-four QTLs to eight chromosomes. According to the recombination nodule frequency map the assigned QTLs are in high recombination frequency regions of the corresponding chromosomes [11]. Diseases caused by different bacteria, fungus and viruses tremendously attenuate yield potential of tomato. Resistance loci associated with diseases caused by Tomato yellow leaf curl virus (*Ty1/3*), *Pseudomonas syringae* (*Pto*), *Xanthomonas campestris* (*Bs4*), *Cladosporium fulvum* (*Cf-9*), Verticillium albo-atrum (*Ve*) and root knot nematode (*Mi*) were identified by map-based cloning (32–37). Physical locations of these pathogen-resistance loci, as derived from their marker or gene sequences, were also shown on the map as references.

## Conclusion

Tomato genome sequence has created a huge impact on *Solanaceae* research. The reference genome is increasingly being used for sequence-based approaches to answer basic biological questions of plant development as well as for agricultural improvement of fruit quality and quantity. Although several databases for tomato in addition to SGN exist, we tried to provide the most sought-after basic information about a genome in TGRD in a user-friendly way and we feel that graphical presentation of this important genomic information will facilitate a better use of tomato genome. A tutorial with graphical and video description has been provided for better use of the site. Regulation of gene expression is equally important as the gene sequence in influencing traits of an organism. For this reason we provided 2 kb-long 5′-upstream sequences with all the genes. Incorporation of SSR- and SNP-information within this region will be useful in exploiting intra- and inter-specific polymorphic sequences in studying functional divergence of homologous genes. Focus of the breeding programs carried out in the last century has been on increase in yield while fruit quality traits did not receive proper importance [47]. Improvement in traits such as, flavor, taste and nutrient content is challenging because these are regulated by biochemically complex processes. Recent advances in mapping some of the QTLs for these traits will lead to our understanding of these complex traits. Availability of tomato genome sequence and effort to assign physical locations of some of the flavor-related QTLs on the reference genome integrated with SSRs and SNPs in TGRD may facilitate biochemical characterization of these traits. Transcriptome sequences of a few wild relatives of tomato are now available [48]. We will keep on updating our site by incorporating SNP data of those wild accessions once their genome sequence data are available to allow robust mining of SNPs with stringent filtering criteria.

## Supporting Information

Figure S1 **Description of schema used to construct tomato genome database.**
(TIF)

Figure S2 **Graphical distribution of frequency of genomic (in black) and genic (in red) SNPs between Heinz1706 and LA1589 in each million base interval of each chromosome.**
(TIF)

Figure S3 **Graphical distribution of frequency of genic SNPs (in red) between Heinz1706 and LA1589 and genes (in black) in each million base interval of each chromosome.**
(TIF)

Table S1 **Chromosome-wise and motif-wise distribution of simple sequence repeats in tomato genome.**
(XLS)

Table S2 **Genes preferentially/specifically expressed in each tissue sample, as compared to others in a tissue-by-tissue comparison.** Genes showing at least two-fold change (upregulated above the blank cell and downregulated below the blank cell) as compared to the other tissue samples were given.
(DOC)

Table S3 **Chromosome-wise distribution and frequency of single nucleotide polymorphic sites between** ***S. lycopersicum*** **Heinz1706 and** ***S. pimpinellifolium*** **LA1589.**
(XLS)

Table S4 **Genomic regions showing high frequency of SNP per gene in 1 Mb interval between** ***S. lycopersicum*** **Heinz1706 and** ***S. pimpinellifolium*** **LA1589.**
(XLS)

Table S5 **A list of genes containing ten or more SNPs (between** ***S. lycopersicum*** **Heinz1706 and** ***S. pimpinellifolium*** **LA1589).**
(XLS)

Table S6 **Structural variations between** ***S. lycopersicum*** **Heinz1706 and** ***S. pimpinellifolium*** **LA1589 in the 5′-upstream region of gene ‘Cell number regulator 1′ (Solyc02g090730.2.1).**
(XLS)
