# Supplementary figures and images for: Tomato Genomic Resources Database: An Integrated Repository of Useful Tomato Genomic Information for Basic and Applied Research
Source: PLoS One. 2014 Jan 21;9(1):e86387. doi: 10.1371/journal.pone.0086387 (PMC3897720; doi:10.1371/journal.pone.0086387)

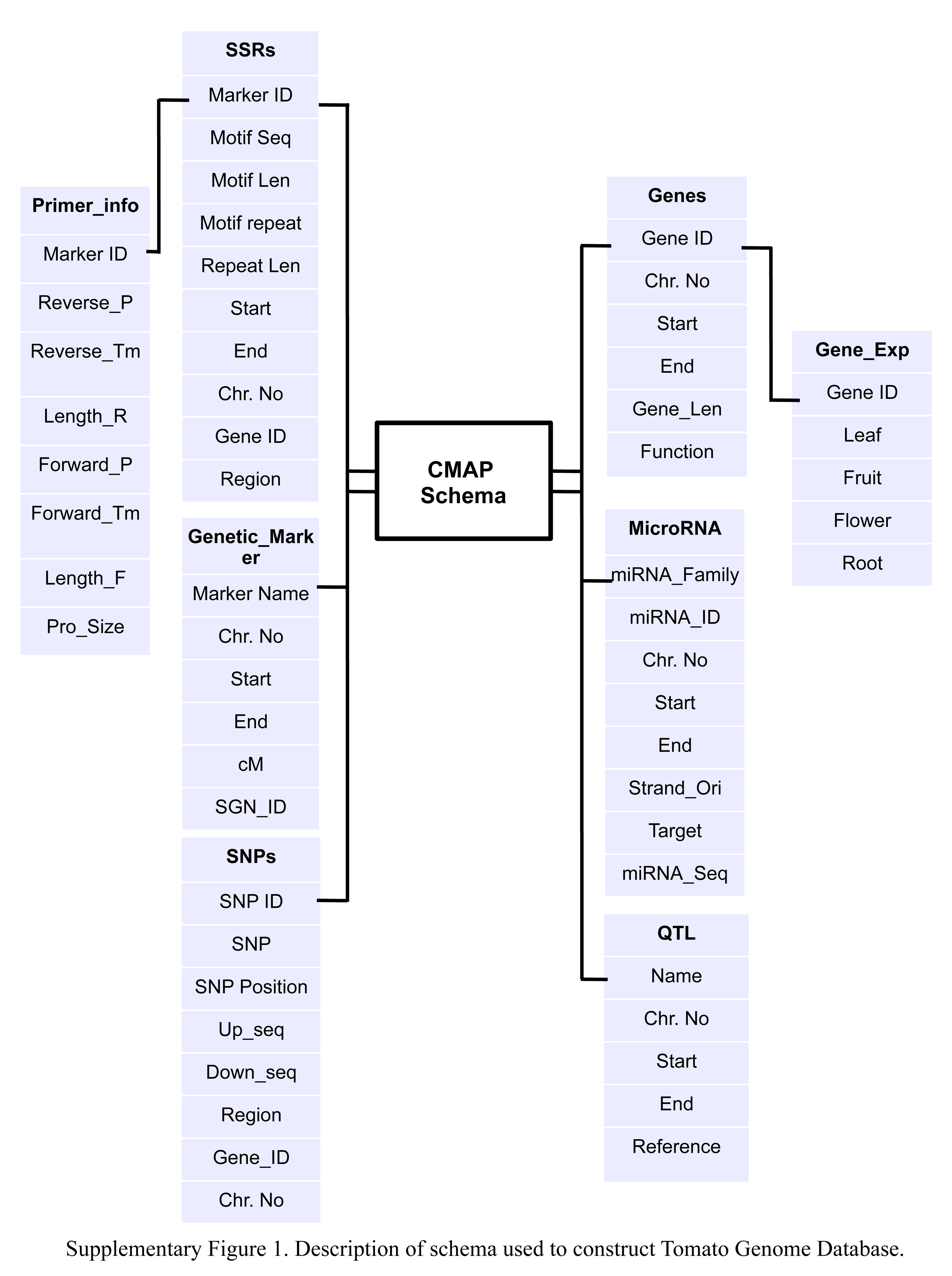

Supplement: Figure S1 — Description of schema used to construct tomato genome database. (TIF) [file pone.0086387.s001.tif]

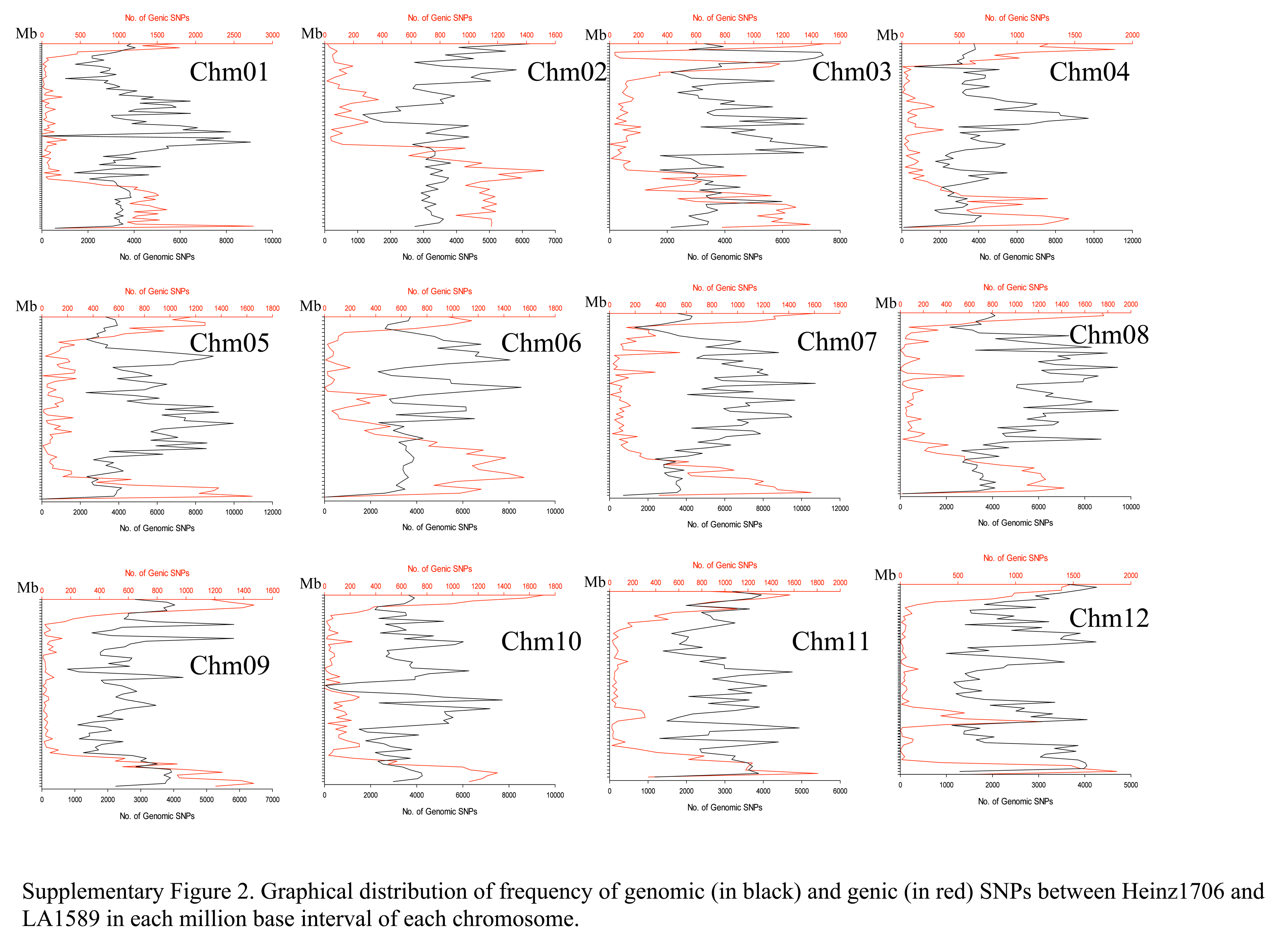

Supplement: Figure S2 — Graphical distribution of frequency of genomic (in black) and genic (in red) SNPs between Heinz1706 and LA1589 in each million base interval of each chromosome. (TIF) [file pone.0086387.s002.tif]

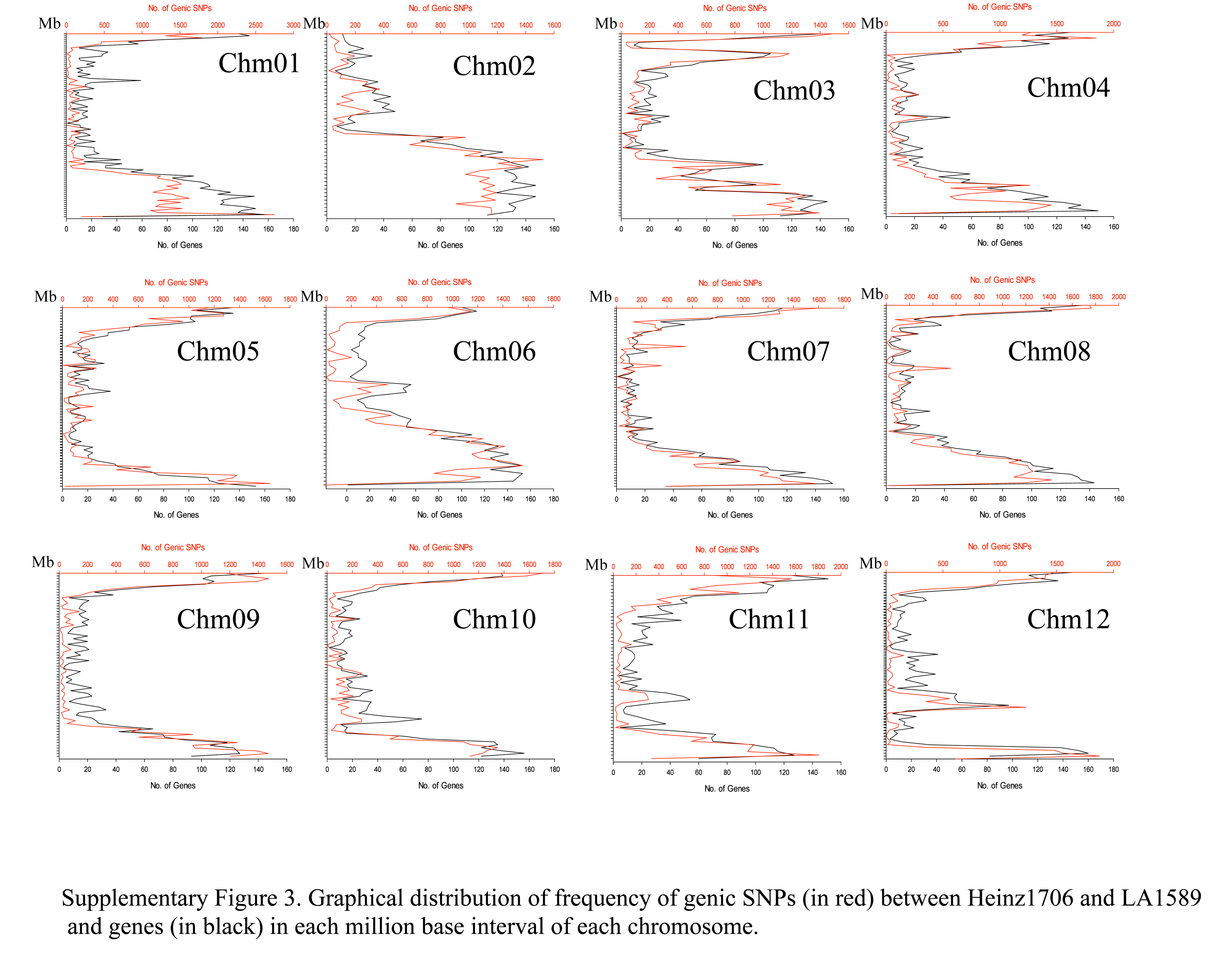

Supplement: Figure S3 — Graphical distribution of frequency of genic SNPs (in red) between Heinz1706 and LA1589 and genes (in black) in each million base interval of each chromosome. (TIF) [file pone.0086387.s003.tif]
