# Supplementary material for: Tomato Genomic Resources Database: An Integrated Repository of Useful Tomato Genomic Information for Basic and Applied Research
Source: PLoS One. 2014 Jan 21;9(1):e86387. doi: 10.1371/journal.pone.0086387 (PMC3897720; doi:10.1371/journal.pone.0086387)
Supplement: Table S2 — Genes preferentially/specifically expressed in each tissue sample, as compared to others in a tissue-by-tissue comparison. Genes showing at least two-fold change (upregulated above the blank cell and downregulated below the blank cell) as compared to the other tissue samples were given. (DOC) [file pone.0086387.s005.doc]

Supplementary Table 2. Genes preferentially/specifically expressed in each tissue sample, as compared to others in a tissue-by-tissue comparison. Genes showing at least two-fold change (upregulated above the blank cell and downregulated below the blank cell) as compared to the other tissue samples were given.

|  | **Leaf** | **Root** | **Flower** | **Mature green fruit** |
| --- | --- | --- | --- | --- |
| **Leaf** |  | 145 | 166 | 183 |
| **Root** | 283 |  | 202 | 191 |
| **Flower** | 225 | 220 |  | 177 |
| **Mature green fruit** | 217 | 153 | 179 |  |
